# Clinical Implications of Suspended Scattering Particles in Motion Observed by Optical Coherence Tomography Angiography

**DOI:** 10.1038/s41598-019-55606-9

**Published:** 2020-01-08

**Authors:** Jaemoon Ahn, Sangheon Han, So Min Ahn, Seong-Woo Kim, Jaeryung Oh

**Affiliations:** 1 Department of Ophthalmology, CHA Bundang Medical Center, CHA University, Seongnam, Republic of Korea; 2 Department of Chemistry, University of Illinois at Urbana-Champaign, Champaign, USA; 3 Department of Ophthalmology, Korea University College of Medicine, Seoul, Republic of Korea

**Keywords:** Predictive markers, Predictive markers

## Abstract

The objective of this study was to investigate the relationship between suspended scattering particles in motion (SSPiM) in optical coherence tomography angiography (OCTA) and treatment response in diabetic macular edema (DME). We retrospectively reviewed the medical records of patients diagnosed with DME who had undergone intravitreal injection. The optical density ratio (ODR) of the intraretinal cyst and the numbers of hyperreflective foci from OCT images and SSPiM from OCTA images were compared, and their association with treatment response was analyzed. Forty-five eyes from 45 patients were included in this study. Twenty-four patients were treated with anti-vascular endothelial growth factor, and 21 patients were treated with a steroid. Binary logistic regression model showed that SSPiM in OCTA images was associated with hyperreflective foci numbers (*P* = 0.038) and mean ODR of the intraretinal cyst (*P* = 0.006). Linear regression model showed that SSPiM in the inner nuclear layer was related to treatment response (*P* = 0.006). SSPiM on OCTA images is related to the poor structural response to treatment in DME.

## Introduction

Optical coherence tomography angiography (OCTA) is a recently developed imaging technique that can visualize blood vessels with depth resolution^1^. The characteristics of OCTA imaging in various diseases have been described^2–7^. OCTA images are obtained using differences in reflectivity from repeated optical coherence tomography (OCT) B scan images^8,9^. Since red blood cells have high reflectivity in OCT images, OCTA images can show the blood vessels.

Because OCTA does not use dye, it is comparatively simple and does not selectively mark blood vessels, unlike other angiography techniques. Therefore, there is the possibility of various artifacts in OCTA images, as have been reported in previous studies^10–13^. However, nonvascular decorrelation signals observed on OCTA are not always artifacts. Kashani *et al*. recently reported a novel artifact-like feature called suspended scattering particles in motion (SSPiM)^14^. Supplementary Figure shows examples of SSPiM. This likely represents actual flow of suspended particles in intraretinal fluid, not an artifact. The authors suggested that more severe blood-retinal barrier (BRB) breakdown resulted in greater particle outflow, leading to SSPiM. We suspect that a difference in BRB breakdown will affect treatment response and be of clinical relevance.

In diabetic patients, the inner BRB is destroyed by disruption of elements maintaining the inner BRB, including occludin of the retinal vascular endothelium, Muller cells, and astrocytes^15^. Breakdown of the inner BRB increases serum exudation from the retinal vessel. Diabetic macular edema (DME) caused by increased exudation is a typical cause of SSPiM. The purpose of the current study is to investigate the relationship between SSPiM and treatment response in DME.

## Results

### Differences According to the Presence or Absence of SSPiM in OCTA Images

Table 1 shows the characteristics of the patients according to the presence or absence of SSPiM in OCTA images. There were no differences in underlying disease, HbA1c, treatment agent, cyst location, cyst size, or the standard deviation of optical density ratio (ODR) between the two groups. However, the mean ODR was greater (5.41 ± 3.26, 2.30 ± 1.46, *P* < 0.001) in the group with SSPiM. The number of hyperreflective foci (HRF) was greater in the group with SSPiM (4.57 ± 3.59, 1.50 ± 3.21, *P* = 0.003). In the group with SSPiM, the size of the intraretinal cyst decreased less after treatment (30.06 ± 54.05%, 69.00 ± 45.46%, *P* = 0.030).

**Table 1:** General characteristics of patients with diabetic macular edema.

	Total (n = 45)	Presence of SSPiM (n = 21)	Absence of SSPiM (n = 24)	*P* value*
Age (years)	58.18 ± 13.78	59.24 ± 10.86	57.25 ± 16.09	0.626^†^
**Underlying disease**				
Hypertension (n, %)	29 (64.44)	16 (76.19)	13 (54.17)	0.212^‡^
Hyperlipidemia (n, %)	9 (20.00)	4 (19.05)	5 (20.83)	1.000^‡^
CKD (n, %)	10 (22.22)	5 (23.81)	5 (20.86)	1.000^‡^
CVA/MI (n, %)	4 (8.89)	2 (9.52)	2 (8.33)	1.000^‡^
Hb_A1c_	7.72 ± 1.64	8.27 ± 1.91	7.16 ± 1.13	0.058^†^
**Injection**				0.767^‡^
Anti-VEGF (n)	24	12	12	
Steroid (n)	21	9	12	
**Location of the cyst**				0.226^‡^
INL (n)	15	9	6	
ONL (n)	30	12	18	
Size of cyst (mm^2^)	0.13 ± 0.09	0.14 ± 0.10	0.12 ± 0.08	1.269^§^
Mean ODR in cyst	3.75 ± 2.90	5.41 ± 3.26	2.30 ± 1.46	<0.001^†^
SD of ODR in cyst	1.72 ± 1.20	2.14 ± 1.48	1.35 ± 0.73	0.105^†^
Hyperreflective foci (n)	2.93 ± 3.33	4.57 ± 3.59	1.50 ± 3.21	0.003^§^
Treatment response (%)	50.83 ± 52.86	30.06 ± 54.05	69.00 ± 45.46	0.030^§^

*P values from multiple comparisons of a single OCT image were adjusted using Bonferroni’s method. Adjusted *P* values were raw *P* values multiplied three, because three factors were measured: the size of the cyst, ODR of the cyst, and the number of hyperreflective foci.

^†^Independent t-test.

^‡^Fisher’s exact test.

^§^Mann-Whitney U test.

INL = inner nuclear layer, OCTA = optical coherence tomography angiography, ODR = optical density ratio, ONL = outer nuclear layer, SD = standard deviation, SSPiM = suspended scattering particles in motion, VEGF = vascular endothelial growth factor.

### Treatment response of intraretinal cysts

Table 2 shows the treatment response of intraretinal cysts observed on OCT image. The proportion of complete response was lower in cysts with SSPiM (0.33 ± 0.39, 0.68 ± 0.35, *P* = 0.017). The proportion of partial response did not vary with or without SSPiM (0.33 ± 0.39, 0.20 ± 0.26, *P* = 0.815). The proportion of no response was higher in cysts with SSPiM (0.33 ± 0.45, 0.11 ± 0.25, *P* = 0.038).

**Table 2:** Treatment response of intraretinal cysts observed on optical coherence tomography image.

	Intraretinal cysts with SSPiM	Intraretinal cysts without SSPiM	*P* value*
Number	2.57 ± 2.09	9.55 ± 7.31	0.003
Proportion of complete response	0.33 ± 0.39	0.68 ± 0.35	0.017
Proportion of partial response	0.33 ± 0.39	0.20 ± 0.26	0.815
Proportion of no response	0.33 ± 0.45	0.11 ± 0.25	0.038

^*^Mann-Whitney U test.

SSPiM = suspended scattering particles in motion.

### Factors related to SSPiM

Table 3 shows the results of univariable and multivariable binary logistic regression analysis of factors related to SSPiM. Multivariable binary logistic regression analysis of factors related to the SSPiM showed that the number of HRF (OR = 1.457, CI = 1.066–1.992, *P* = 0.038) and the mean ODR of the intraretinal cyst (OR = 2.287, CI = 1.150–4.547, *P* = 0.006) were significant variables.

**Table 3:** Univariable binary logistic regression analysis of factors related to the suspended scattering particles in motion.

Parameters	Univariable analysis				Multivariable analysis			
	Odds ratio	CI (lower)	CI (upper)	*P* value	Odds ratio	CI (lower)	CI (upper)	*P* value
Hyperreflective foci	1.483	1.119	1.965	0.006	1.457	1.066	1.992	0.038*
Location of the cyst (ONL)	2.250	0.635	7.973	0.209				
Cyst size	1.000	1.000	1.000	0.475				
Mean ODR in cyst	1.852	1.234	2.779	0.003	2.287	1.150	4.547	0.006*
SD of ODR in cyst	2.000	1.015	3.938	0.045	0.514	0.134	1.973	0.332
Central macular thickness	0.998	0.993	1.003	0.423				

CI = confidence interval, ODR = optical density ratio, ONL = outer nuclear layer, SD = standard deviation.

### Factors related to treatment response

Among the 45 patients with DME, 24 patients were injected with anti-vascular endothelial growth factor (VEGF) and 21 patients were injected with a steroid. Table 4 shows results of univariable linear regression analysis of factors related to treatment response. Linear regression analysis revealed that treatment response was related to only SSPiM in the inner nuclear layer (INL) (β = −0.457, *P* = 0.006).

**Table 4:** Univariable linear regression analysis of factors related to the treatment response.

Parameters	Total (n = 45)		Anti-VEGF (n = 24)		Steroid (n = 21)	
	β	P value	β	P value	β	P value
Hyperreflective foci	0.248	0.100	0.288	0.172	0.205	0.373
Location of the cyst (ONL)	0.149	0.991	0.249	0.241	0.084	0.717
Cyst size	−0.109	0.474	0.210	0.325	−0.337	0.135
Mean ODR in cyst	0.162	0.288	0.049	0.818	0.310	0.171
SD of ODR in cyst	0.033	0.827	−0.041	0.848	0.094	0.687
SSPiM in INL	−0.457	0.006	−0.477	0.039	−0.427	0.099
SSPiM in ONL	−0.350	0.130	−0.164	0.673	−0.773	0.005

INL = inner nuclear layer, ODR = optical density ratio, ONL = outer nuclear layer, SD = standard deviation, SSPiM = suspended scattering, VEGF = vascular endothelial growth factor.

The analysis of patients treated with anti-VEGF showed that treatment response was related to SSPiM observed in the INL (β = −0.477, *P* = 0.039). The analysis of patients treated with a steroid showed that treatment response was related to SSPiM observed in the ONL (β = −0.773, *P* = 0.005).

### Factors related to visual acuity and central macular thickness

Table 5 shows the results of univariable linear regression analysis of factors related to visual acuity and central macular thickness. OCT and OCTA image factors were not related to visual acuity in this study. Central Macular Thickness(CMT) was related to sex (β = −0.326, *P* = 0.029), cyst size (β = 0.552, *P* < 0.001), mean ODR in cyst (β = −0.299, *P* = 0.046), and the SD of the ODR in the cyst (β = −0.311, *P* = 0.037). The reduction in CMT was related to cyst size (β = 0.307, *P* = 0.040), mean ODR in cyst (β = −0.296, *P* = 0.048), SSPiM in INL (β = −0.440, *P* = 0.008), and SSPiM in outer nuclear layer (ONL) (β = −0.357, *P* = 0.021).

**Table 5:** Univariable linear regression analysis of factors related to visual acuity and central macular thickness.

	Baseline VA		Visual gain		CMT		Reduction in CMT	
	β	P value	β	P value	β	P value	β	P value
Sex (female)	0.410	0.211	0.247	0.464	−0.326	0.029	−0.182	0.230
Age	0.425	0.193	0.435	0.181	−0.075	0.627	−0.027	0.861
Injection (steroid)	0.389	0.237	0.392	0.233	0.007	0.961	0.128	0.403
Hyperreflective foci	0.170	0.617	−0.043	0.900	−0.002	0.991	−0.144	0.344
Cyst size	0.331	0.320	0.097	0.776	0.522	<0.001	0.307	0.040
Mean ODR in cyst	−0.170	0.618	0.013	0.971	−0.299	0.046	−0.296	0.048
SD of ODR in cyst	0.135	0.693	0.028	0.934	−0.311	0.037	−0.233	0.124
SSPiM in INL	−0.053	0.727	−0.099	0.571	−0.117	0.502	−0.440	0.008
SSPiM in ONL	−0.194	0.203	−0.419	0.066	−0.288	0.231	−0.385	0.104
Baseline VA	—	—	0.850	0.001	0.370	0.012	0.372	0.012

CMT = central macular thickness, INL = inner nuclear layer, ODR = optical density ratio, ONL = outer nuclear layer, SD = standard deviation, SSPiM = suspended scattering particles in motion, VA = visual acuity.

### Case 1

**Figure 1:**
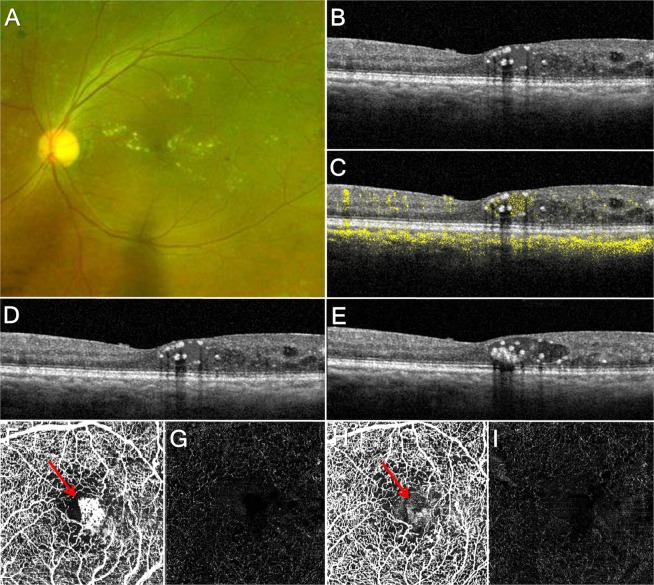
A patient with diabetic macular edema who underwent intravitreal triamcinolone injection. (**A**) Fundus photograph. (**B**) Optical coherence tomography (OCT) image. There is an intraretinal cyst with hyperreflective foci. (**C**) OCT image with optical coherence tomography angiography (OCTA) signal of the intraretinal cyst. (**D**) OCT image before treatment of an intraretinal cyst 0.07 mm^2^ in size. (**E**) OCT image one month after intravitreal triamcinolone injection; the largest intraretinal cyst remains and is 0.09 mm^2^ in size. Small intraretinal cysts without OCTA signal have disappeared. (**F**) OCTA image including inner nuclear layer (INL) with suspended scattering particles in motion (SSPiM) before treatment (arrow). (**G**) OCTA image including outer nuclear layer (ONL) before treatment without SSPiM. (**H**) OCTA image including INL after treatment with decreased SSPiM (arrow). (**I**) OCTA image including ONL after treatment without SSPiM.

A 50-year-old woman visited the hospital for diabetic retinopathy screening. She had been treated for diabetes and hypertension over the prior 3 years. On examination, she was diagnosed with very severe nonproliferative diabetic retinopathy in the right eye and proliferative diabetic retinopathy in the left eye. She underwent panretinal photocoagulation in both eyes. One year later, she complained of decreased vision in both eyes. She was diagnosed with DME in both eyes, and treated with focal laser therapy. DME disappeared, but recurred in the left eye seven months later. On OCT and OCTA, there was an intraretinal cyst with SSPiM and HRF (Fig. 1). After 4 weeks of intravitreal triamcinolone injection, the size of the largest cyst remained unchanged. Another cyst without SSPiM also remained unchanged. However, three smaller cysts without SSPiM disappeared.

### Case 2

**Figure 2:**
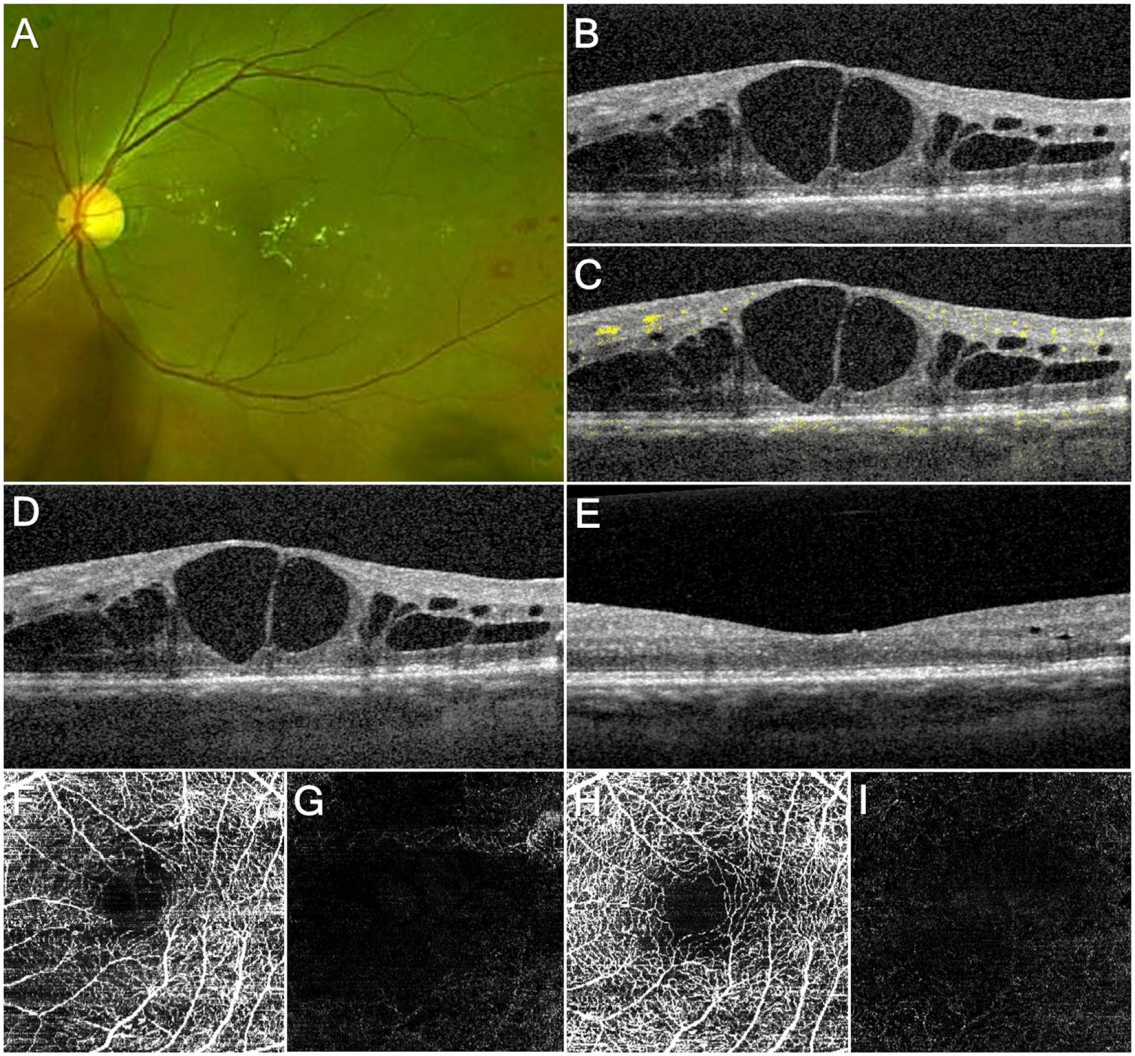
A patient with diabetic macular edema who underwent intravitreal ranibizumab injection. (**A**) Fundus photograph. (**B**) Optical coherence tomography (OCT) image. There are several intraretinal cysts. (**C**) OCT image with optical coherence tomography angiography (OCTA) signal; the intraretinal cysts do not show OCTA signal. (**D**) OCT image before treatment; the largest intraretinal cyst size is 0.15 mm^2^. (**E**) OCT image one month after intravitreal triamcinolone injection; the intraretinal cysts have disappeared. (**F**) OCTA image including inner nuclear layer (INL) without suspended scattering particles in motion (SSPiM) before treatment. (**G**) OCTA image including outer nuclear layer (ONL) without SSPiM before treatment. (**H**) OCTA image including INL after treatment without SSPiM. (**I**) OCTA image including ONL including ONL after treatment without SSPiM.

A 49-year-old man presented with decreased vision in the left eye. He had been treated for diabetes and hypercholesterolemia for 9 years. He also underwent panretinal photocoagulation in the nasal area at another hospital 4 years ago. On examination, he was diagnosed with very severe nonproliferative diabetic retinopathy in both eyes and DME in the left eye (Fig. 2). On OCT and OCTA, there were several intraretinal cysts without SSPiM. After 4 weeks of intravitreal ranibizumab injection, the intraretinal cysts disappeared.

### Case 3

**Figure 3:**
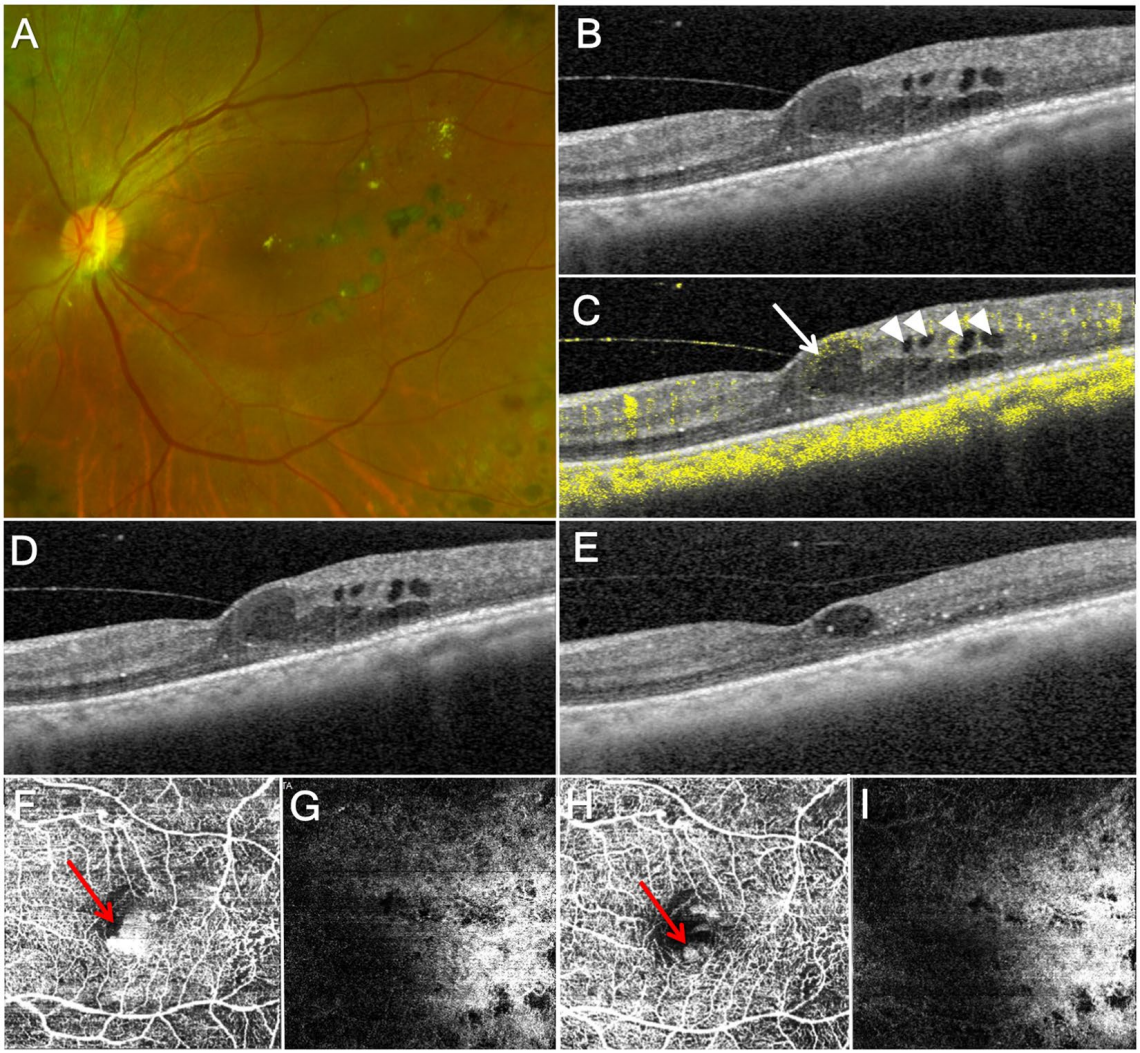
A patient with diabetic macular edema who underwent intravitreal triamcinolone injection. (**A**) Fundus photograph. (**B**) Optical coherence tomography (OCT) image. There are several intraretinal cysts. (**C**) OCT image with optical coherence tomography angiography (OCTA) signal. The largest intraretinal cyst (arrow) has OCTA signal, while other intraretinal cysts do not have OCTA signal (arrowhead). (**D**) OCT image before treatment; the largest intraretinal cyst is 0.04 mm^2^ in size. (**E**) OCT image one month after intravitreal triamcinolone injection; the largest intraretinal cyst size is 0.02 mm^2^, a reduction of 48.14%. (**F**) OCTA image including inner nuclear layer (INL) with suspended scattering particles in motion (SSPiM) before treatment (arrow). (**G**) OCTA image including outer nuclear layer (ONL) without SSPiM before treatment. (**H**) OCTA image including INL with decreased SSPiM after treatment (arrow). (**I**) OCTA image including ONL without SSPiM after treatment.

A 55-year-old woman presented with decreased vision in the left eye. She had been treated for the prior 19 years for diabetes and hypertension. On examination, she was diagnosed with high risk proliferative diabetic retinopathy in both eyes and DME in the left eye. She underwent panretinal photocoagulation in both eyes and received two monthly intravitreal injections of bevacizumab in the left eye. There was no response to bevacizumab injection, but DME was improved after focal laser treatment. One year later, DME recurred (Fig. 3). On OCT and OCTA, there were one intraretinal cyst with SSPiM and several small intraretinal cysts without SSPiM. After 4 weeks of intravitreal triamcinolone injection, the SSPiM-free intraretinal cysts disappeared, while the SSPiM-positive intraretinal cysts persisted.

## Discussion

In this study, we imaged DME using OCT and OCTA. We assessed the intraretinal cystic cavity in OCT images and evaluated the relationship between OCT findings and SSPiM in the intraretinal cysts in OCTA images. The greater the ODR of the intraretinal cyst and the greater the number of HRF, the greater the probability of observing SSPiM. The results of this study are very similar to a previous study^14^. The BRB prevents the migration of large molecules, such as serum proteins, under normal conditions. Increased VEGF under ischemic conditions, like DME, weakens BRB function^16^. This allows serum proteins to permeate the retinal interstitium. Previous studies have shown that proteins, such as albumin or serum reactants, in the intraretinal cyst are hyperreflective in OCT images^17^. Because OCTA does not selectively highlight blood vessels, unlike other angiography techniques, a material moving at a rate similar to blood flow can be observed in OCTA.

OCTA is known to detect flows between 0.3 and 4 mm per second. It has been previously reported that OCTA does not enable detection of retinal capillary flow of less than 0.3 mm per second or turbulent blood flow inside microaneursms^18,19^. However, this study suggests that other factors besides the flow velocity may influence OCTA images. Univariable binary logistic regression revealed that the standard deviation of the intraretinal cyst ODR was related to SSPiM in OCTA images. The presence of hyperreflective materials, such as HRF, increases the standard deviation of ODR. A particle with high reflectivity can cause OCTA image artifacts, like a false positive decorrelation in a patient with pigment epithelial detachment^10^. Another study has reported that a large lumen, like the intraretinal cysts in this study, induced multiple dynamic backscattering and showed a OCTA signal differing from the capillary^20^. We hypothesize that a flow faster than 0.3 mm per second or hyperreflective materials in the intraretinal cyst can be observed in OCTA as SSPiM.

The number of HRF was correlated with the SSPiM in OCTA. HRF in the intraretinal cyst are also found in severe BRB disruption^21^. Therefore, HRF and SSPiM may be related because both appear to occur when the BRB breaks down. Meanwhile, another study using OCTA had reported that HRF, especially in the Henle’s fiber layer, had a decorrelation signal in OCTA images^22^. With HRF, SSPiM is more likely to be observed in OCTA images, but this cannot be regarded as reflecting the entire SSPiM in the intraretinal cyst.

In this study, we analyzed the relationship between the SSPiM in OCTA images and treatment response as a structural outcome. The presence of SSPiM in OCTA images was associated with poor treatment response. ODR and HRF, as related to SSPiM in this study, were related to treatment response in previous OCT-based studies. The intraretinal fluid turbidity in OCT images, corresponding to the ODR in this study, could influence the treatment response^23^. The absence of HRF was associated with good treatment response^24^. When SSPiM was divided into layers, SSPiM observed in the INL was most greatly related to treatment response; whereas, SSPiM observed in the ONL was not related to treatment response. On the other hand, in cases when the cyst exists in the ONL, the developmental mechanism may differ from the mechanism in the inner layer. Bringmann *et al*. reported that retinal edema in outer retina may not be accompanied by vascular leakage and may be explained by a disturbance in dehydrating function^25^. Therefore, SSPiM in the ONL may not be related to fluid efflux from retinal vessels, and therefore may be considered independent of treatment response.

We divided the groups according to treatment modality and analyzed factors predicting treatment response. Similar to the results for the entire patient population, SSPiM in the INL of the OCTA images was associated with poor treatment response in the anti-VEGF group. However, treatment response was not associated with SSPiM in the INL, but was associated SSPiM in the ONL in the steroid group. Steroids have a stronger effect than anti-VEGF agents, acting on the non-VEGF pathway as well as on the VEGF pathway that causes DME^26^. There is a tendency to administer steroids in patients who are predicted to have poor treatment response in a clinical setting. This study had a small number of patients and retrospective study design, and thus could not determine whether steroid or anti-VEGF treatment is more effective for patients with SSPiM on OCTA because of bias. However, we did show that both steroid and anti-VEGF treatments were less effective in patients with SSPiM on OCTA. A prospective study with a randomized and controlled design is needed to clarify the differences between treatment regimens.

In general, CMT is used as an indicator of anatomical changes related to DME treatment, while visual acuity is used as an indicator of functional changes. In this study, SSPiM was related to treatment response and change in CMT. However, cyst size and mean ODR of the cyst were related only to changes in CMT. In previous studies, intraretinal cysts have been reported as negative predictors of functional improvement^27,28^. Therefore, SSPiM is expected to be related to visual acuity or visual improvement after treatment. However, the parameters of OCT and OCTA images were not related to visual acuity in this study. We did not evaluate the cyst in the foveal center. The largest intraretinal cyst analyzed in this study was often located in the foveal center, and the change in CMT showed similar results to treatment response. It was not necessarily located at the foveal center, so there was no exact match. The results of this study alone cannot clarify the relationship between SSPiM and visual acuity or between SSPiM and change in CMT.

This study has several limitations. First, it utilized a retrospective design with a relatively small number of cases. Statistical significance should be interpreted with caution, especially in the multivariable regression model. Second, segmentation error was not corrected. The presence of an intraretinal cyst increases the probability of an automated segmentation error. This can cause errors in the intraretinal cyst position, but it has the advantage of eliminating bias due to manual measurements and of quick judgements. Rapid assessment based on automatically segmented images in the clinical environment is useful. Third, the processing algorithm differs from machine to machine in OCTA. The OCTA in this study used a full-spectrum amplitude-decorrelation method. Although full-spectrum amplitude-decorrelation has an advantage of good axial resolution, it has the disadvantage of noise in comparison to split-spectrum amplitude-decorrelation^29–31^. It is possible that studies using other OCTA machines may obtain different results. Finally, we analyzed only the largest cysts in each subject, since there may be differences in the presence or absence of SSPiM in each cyst. The results of this study thus can only reflect the treatment response of individual cysts, and not of the patient. Since the largest cyst is not always located in the macular center, treatment response in this study may vary from treatment response with regard to DME or visual acuity.

In conclusion, SSPiM on OCTA images is related to a poor structural response to treatment. Thus, we believe that our findings could provide some information on the characteristics and treatment response of intraretinal cysts. Lastly, these results suggest the possibility of using SSPiM as a biomarker for DME.

## Methods

### Ethics statement

The Institutional Review Board of Korea University approved this retrospective case series study, waiving the requirement for informed consent for study participation. All research and data collection were conducted in accordance with the tenets of the Declaration of Helsinki by the World Medical Association.

### Patients

We reviewed the medical records of consecutive patients who were diagnosed with DME and who had undergone intravitreal injection between March 2017 and February 2018 at the Korea University Medical Center. Exclusion criteria were as follows: (1) other ocular diseases, such as retinal vein occlusion; (2) a history of intraocular treatment within 6 months (e.g. intravitreal injection, laser photocoagulation, or cataract surgery); (3) a history of vitrectomy; (4) OCTA images with artifacts not related to the intraretinal cyst, such as blinking artifacts, or motion artifacts; and (5) OCTA images with poor quality (signal strength index of less than 30).

Patients were treated with a steroid or anti-VEGF, and treatment modalities were chosen by one retinal specialist. Patients with pseudophakia or difficulty visiting the hospital were treated with a steroid, and those with a history of previous elevation of intraocular pressure were treated with anti-VEGF. Treatment response was evaluated 4 weeks after intravitreal injection. For patients who underwent multiple intravitreal injections, the images taken at the first intravitreal injection during the study period were analyzed.

### Image acquisition

The OCTA instrument (Heidelberg Spectralis OCT2, Family acquisition module 6.4.21.0; Heidelberg Engineering, Heidelberg, Germany) used in this study had a central wavelength of 870 nm, speed of 85,000 A-scans/second, horizontal resolution of 5.7 μm, and an axial resolution of 3.9 μm. An OCTA scan pattern was 10 × 10 degrees (3 × 3 mm; consisting of 512 B scans) centered on the macula. The OCTA acquisition software utilized a full-spectrum and probabilistic algorithm. *En face* OCTA images were exported from image viewer software (Heidelberg Eye Explorer, software version 1.21; Heidelberg Engineering, Heidelberg, Germany) after application of projection artifact removal in the deeper layers.

### Location and size of intraretinal cyst and treatment response

The largest intraretinal cyst observed within a 3 × 3 mm area centered on the macula was used for analysis. CMT was automatically measured on an *en face* macular map. OCT images were analyzed using B scan images through the longest horizontal diameter of the intraretinal cyst. The actual location of the intraretinal cyst, not the automatically detected location, was determined by agreement of two researchers (JA and SH). In cases of disagreement, a final determination was made after review by a third observer (SWK).

The size of intraretinal cyst was measured from OCT images using ImageJ software (National Institutes of Health; http://imagej.nih.gov/ij/) with a freehand selection tool. This measurement was performed before and 4 weeks after treatment. Treatment response was determined by comparing OCT images before and after treatment and defined as follows: $\frac{{\rm{cyst}}\,{\rm{size}}\,{\rm{before}}\,{\rm{treatment}}-{\rm{cyst}}\,{\rm{size}}\,{\rm{after}}\,{\rm{treatment}}}{{\rm{cyst}}\,{\rm{size}}\,{\rm{before}}\,{\rm{treatment}}}$. Two observers (JA and SH) were masked to patient information and independently measured the area of the intraretinal cyst. Interobserver agreement for the area of the intraretinal cyst was satisfactory (intraclass correlation coefficients of 0.956).

#### Treatment response of intraretinal cysts

Treatment response of intraretinal cysts including the largest cyst was determined by comparing OCT images before and after treatment: (1) complete response, complete loss of intraretinal cysts; (2) partial response, reduced intraretinal cyst size; (3) no response, unchanged or increased intraretinal cyst size. The presence of SSPiM in each cyst was confirmed using OCTA image. The proportion of treatment response was calculated by comparing the number of intraretinal cysts before and after treatment.

### Optical density ratio of intraretinal cyst

The ODR of intraretinal cyst were calculated using ImageJ software. The ODR was measured according to a method used in a previous study with minor modification^32^. In brief, the total intraretinal cyst and vitreous humor were selected. The gray-level intensity of the selected region was measured and ODR was calculated by dividing the gray-level intensity of the intraretinal cyst by the gray-level intensity of the vitreous humor. Two observers (JA and SH) were masked to patient information and independently analyzed ODR. Interobserver agreement for ODR was satisfactory (intraclass correlation coefficients of 0.994).

### Hyperreflective foci and suspended scattering particles in motion

The number of HRF in the OCT images was counted. HRF present in the cyst and in the margins of the cyst were included in the analysis. The presence of SSPiM was confirmed using *en face* OCTA images. The presence of HRF in OCT images and SSPiM in OCTA images were determined by agreement of two observers (JA and SH). In cases of disagreement, a final determination was made after review by a third observer (SWK).

### Statistical analysis

Continuous variables were represented as the mean ± standard deviation and categorical variables were expressed as the count (%). Normal distribution of variables was determined using a Kolmogorov-Smirnov test. General characteristics and parameters were compared with Pearson’s chi-square test or Fisher’s exact test for categorical variables and Mann-Whitney U test or Independent t-test for continuous variables. *P* values from multiple comparisons of a single OCT image were adjusted using Bonferroni’s method.

Linear regression or binary logistic regression analyses were performed to analyze the relationship among the variables. Backward variable elimination method was used to select some important variables in the multivariable regression models including only covariates with a *P* < 0.05 in the univariable logistic regression models. Statistical analyses were performed with SPSS software version 25.0 for Windows (IBM Corp., Armonk, NY, USA). Results were considered statistically significant at *P* values < 0.05.

## Supplementary information

Examples of suspended scattering particles in motion
