# Supplementary material for: Clinical Implications of Suspended Scattering Particles in Motion Observed by Optical Coherence Tomography Angiography
Source: Sci Rep. 2020 Jan 8;10:15. doi: 10.1038/s41598-019-55606-9 (PMC6949280; doi:10.1038/s41598-019-55606-9)
Supplement: Supplementary file 1 — Examples of suspended scattering particles in motion [file 41598_2019_55606_MOESM1_ESM.docx]

**Clinical Implications of Suspended Scattering Particles in Motion Observed by Optical Coherence Tomography Angiography**

Jaemoon Ahn^1^, Sangheon Han^2^, So Min Ahn^3^, Seong-Woo Kim^3^ *, Jaeryung Oh^3^

^1^Department of Ophthalmology, CHA Bundang Medical Center, CHA University, Republic of Korea

^2^Department of Chemistry, University of Illinois at Urbana-Champaign, USA

^3^Department of Ophthalmology, Korea University College of Medicine, Republic of Korea


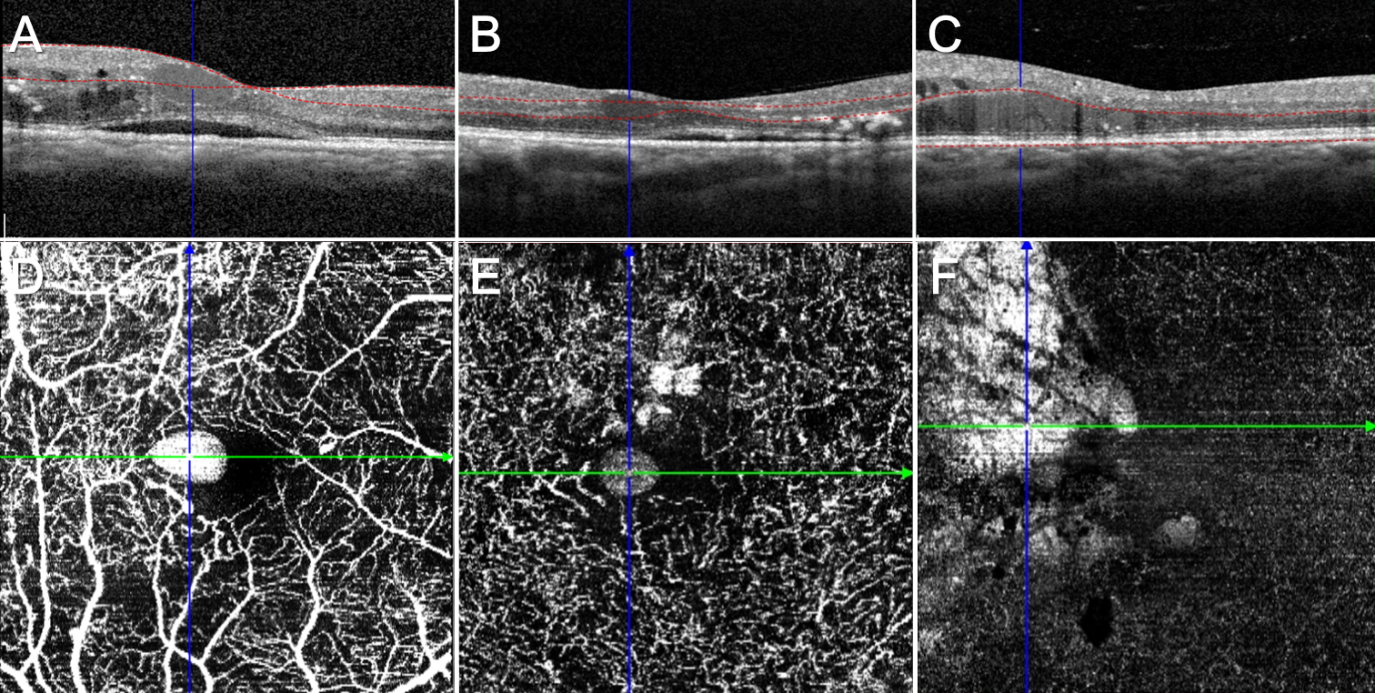


Supplementary Figure. Examples of suspended scattering particles in motion (SSPiM). Upper raw. Optical coherence tomography (OCT) b-scan images. Lower raw. En face OCT angiography images. Left column. Superficial capillary plexus layer. Middle column. Deep capillary plexus layer. Right column. Outer retinal layer.
